# Exploring factors associated with pain in hemodialysis patients: a multicenter cross‐sectional study from Palestine

**DOI:** 10.1186/s12882-021-02305-1

**Published:** 2021-03-17

**Authors:** Maha K. Marzouq, Aseel F. Samoudi, Ahmad Samara, Sa’ed H. Zyoud, Samah W. Al-Jabi

**Affiliations:** 1 Department of Medicine, College of Medicine and Health Sciences, An-Najah National University, 44839 Nablus, Palestine; 2 Poison Control and Drug Information Center (PCDIC), College of Medicine and Health Sciences, An-Najah National University, 44839 Nablus, Palestine; 3 Department of Clinical and Community Pharmacy, College of Medicine and Health Sciences, An-Najah National University, 44839 Nablus, Palestine; 4 Clinical Research Centre, An-Najah National University Hospital, 44839 Nablus, Palestine

**Keywords:** Hemodialysis, Pain, Palestine

## Abstract

**Background:**

Chronic kidney disease (CKD) is a rising medical concern around the world. End-stage kidney disease (ESKD) is the last stage of CKD stages that necessitates renal replacement therapy (RRT), such as hemodialysis (HD), which seems to be the most commonly used type. However, patients on HD still suffer from high mortality and morbidity rates compared to those who receive a kidney transplant. Therefore, we aimed in this study to assess the prevalence of pain among ESKD patients on HD, as well as to explore the factors that were associated with this complaint.

**Methods:**

We conducted a multicenter cross-sectional study in the West Bank, Palestine, between August and November 2018. We used questionnaire-based direct interviews with subjects. After reviewing previous studies in the field, we developed our questionnaire and included items on patients’ social, demographic, and clinical characteristics, including dialysis-related data. It also contained the Brief Pain Inventory (BPI) to assess different aspects of pain symptoms. A convenience sampling technique was used to collect data.

**Results:**

Of the 300 participants, 66.3 % reported having chronic pain. HD sessions themselves were the most commonly cited cause for pain (21.6 %). The most commonly cited site of pain was the upper and lower limbs (37.3 %). Paracetamol was the most frequently used pharmacotherapy for pain alleviation. Multiple regression analysis showed that BMI (p = 0.018), gender (p = 0.023), and the number of comorbidities (p < 0.001) were independently associated with pain severity score.

**Conclusions:**

Pain is a highly prevalent symptom among HD patients in Palestine. Subpopulations with higher pain severity include females, patients with higher BMI, and those with multiple comorbidities. Healthcare providers should routinely assess pain in HD patients as it is considered a significant concern. This would involve pain assessment and development of a treatment plan to improve clinical outcomes. The nephrology associations should also push for pain management in HD patients as a clinical and research priority to improve pain-related disability.

## Background

End-stage kidney disease (ESKD) is associated with increased morbidity and mortality over the world. ESKD is considered the last stage of chronic kidney disease (CKD) that needs renal replacement therapy (RRT) in the form of renal transplantation, peritoneal dialysis, or hemodialysis (HD) [1, 2]. ESKD prevalence and incidence are growing globally. Based on information on 120 countries that have dialysis programs, about almost two million people were on renal replacement therapy by the end of 2005 [3], with 1,297,000 (68 %) of them receiving HD. In Palestine, the overall number of people with HD in the West Bank in 2016 was 1119 patients [4]. This number has increased to 1216 patients in 2017 [5]. In developed countries, diabetic glomerulopathy is responsible for around 44 % of all new ESKD cases [6], followed by hypertensive nephrosclerosis, old age, obesity, and cardiovascular disease, as other notable factors. ESKD can result in kidney failure, cardiovascular disease, and even premature death [1].

HD is the most commonly used type of RRT, with over 90.0 % of patients receiving it [7]. It is also highly effective, halting most of the clinical complications and extending life expectancy [8]. However, patients on HD still suffer from high mortality and morbidity rates due to its complications, including decreased blood pressure, increased blood pressure, nausea, vomiting, vascular access compromise, infections, and chronic pain. These complications require management and necessitate multiple hospital admissions that result in higher healthcare costs and demands on the national level [2, 9]. Pain is a common complication in patients with ESKD and impacts on the quality of life (QOL) [10]. A 2016 systematic review reported the prevalence of chronic pain in ESKD patients on HD, which was up to 82 % and 92 % [11].

The International Association for the Study of Pain (IASP) describes pain as a physical or emotional unpleasant feeling associated with potential or actual harm and may cause weakened memory, inattention, problems with sleep, symptoms of depression, symptoms of anxiety, impotence, diminishing physical activity, and interrupted social relations and duties [12]. Pain could be acute, subacute, or chronic. Chronic pain lacks the warning of acute physiologic nociception function and stays or reoccurs for more than 3 to 6 months [13].

In ESKD patients, three broad categories of pain are usually described: poorly localized, throbbing, cramp-like, deep nociceptive pain; stabbing, aching, paroxysmal, and electric shock-like neuropathic; and mixed pain, which is the presence of both forms of pain at once [14].

Approximately 55 % of HD patients reported extreme pain in the previous 24 h, and 75 % of HD patients reported poor pain control [15]. The reported causes of pain were diverse and included musculoskeletal, the dialysis procedure itself, peripheral polyneuropathy, peripheral vascular disease, polycystic kidney disease, malignancy, carpal tunnel, trauma, osteoarthritis, osteoporosis, and calciphylaxis [16, 17]. Management options include opioids, gabapentin, acetaminophen, tramadol, and nonsteroidal anti-inflammatory drugs [18]. Therefore, drug interaction and confusion in the management plan is not unusual [10]. Poorly controlled pain in HD patients can lead to psychological disturbances, disrupted sleep, reduced compliance with dialysis, and a decline in overall QOL [15, 19].

No previous studies have investigated the prevalence and factors that might be associated with chronic pain in HD patients. It is not obvious whether the situation is comparable to or different from that of other countries [10]. In this study, we aimed to explore the epidemiology of pain among ESKD patients on HD and explore variables that might be correlated with chronic pain and the different therapies used to treat this problem. This study’s results can help achieve an effective management plan and improve the healthcare system outcomes on this front.

## Methods 

### Study design and setting

We conducted a multicenter, cross-sectional study. Its setting was the HD centers at the Governmental Hospitals in Tulkarem, Qalqilya, Jenin, and Tubas, as well as that of An-Najah National University Hospital, from August to November 2018.

### Study population and sampling technique

The Palestinian Ministry of Health reported that eleven dialysis centers provided services to the West Bank population in Palestine, all of which were inside hospital buildings. Their size varied between 1 and 50 dialysis machines. We targeted five dialysis centers located in the Northern West Bank. There were approximately 570 patients served by these units at the time this study was carried out [5]. Raosoft Sample Size Calculator () was used in calculating the sufficient sample size for this study. After setting the margin of error at 5 %, the confidence interval at 95 %, and the population size at 570, the minimum sample size that we needed to include was 220. We used convenience sampling to recruit and interview a total of 330 patients, and 300 of them were accepted to participate in our study distributed as follows: 68 from Tulkarem, 30 from Qalqilya, 82 from Jenin, 18 from Tubas, and 102 from An-Najah National University hospital.

### Data collection instrument

Subjects were interviewed using a questionnaire that consisted of three sections. The structured questionnaire used in this study was based on previously published studies [20–22]. The current research is a secondary analysis of data on pain and HRQoL that was previously published using various approaches in another study [23]. Socio-demographic data, including gender, age, occupation, level of education, type of residence, level of monthly income, and body mass index (BMI), were collected in the first section. We adopted the following categories for BMI: underweight (BMI of < 18.5 kg/m^2^), normal (BMI of 18.5–24.9 kg/m^2^), overweight (BMI of 25-29.9 kg/m^2^), and obese (BMI of ≥ 30 kg/m^2^) [24]. Dialysis-related and other clinical data including dialysis vintage (the duration that the subjects have been receiving HD for in years), weekly frequency, session duration, history of the kidney (for subjects who resumed dialysis due to transplant failure), the total number of chronic comorbidities, and the number of chronically used medications were assessed in the second Secs. [20, 25–27].

The third section contains the validated Arabic version of the Brief Pain Inventory (BPI) scale [22], which is a tool for the evaluation of pain, its intensity and characteristics, and the assessment of its impact on important aspects of a patient’s QOL. Pain severities were determined in the BPI by 0–10 scale items for the worst pain, the least pain, and the average pain felt during the previous 24 h, as well as the current pain. Scores for these four pain severity items were used to calculate the pain severity score, to sum up the numbers to which the subject answers, making the range of the pain severity score 0–40. Pain interference was determined by asking the subjects to rate the effect of pain on seven domains (activity level in general, ability to walk, mood, regular work, ability to sleep, relationships with other people, and enjoying life) on a 0–10 scale and calculating the sum of the scores for all seven items, with the result representing the pain interference score, which therefore ranged from 0 to 70. In addition to these two scores, BPI also assessed pain site, pain relief modalities, and the degree to which pain improved with treatment. We obtained permission from the tool’s developers (i.e., MD Anderson Symptom Tools) at the University of Texas for using the Arabic BPI in this survey.

### Statistical analysis

We used SPSS version 21 for data analysis. Descriptive analysis was used to describe clinical and socio-demographic variables. The Kolmogorov-Smirnov test was used to test the normality for continuous variables. Characteristic variables were represented by their frequencies and percentages, means and standard deviations, and/or medians and interquartile ranges. Kruskal-Wallis H and Mann Whitney U tests were then used to test the associations between these variables and pain scales. A p-value < 0.05 was considered significant. Additionally, to measure the degree of association between pain scales, the Spearman correlation coefficient was applied, while the Cronbach alpha test was used to assess their internal consistency. Finally, multiple linear regression analysis was implemented to identify independently associated variables from those that showed significant association in bivariate testing with pain severity. Any *p*-value of less than 0.05 was considered statistically significant.

## Results

### Demographic and clinical characteristics

Overall, 330 patients with HD were asked to take part in the survey, and 300 of these agreed and were represented in the final study, accounting for a 90.9 % response rate. Participants had a mean age of 54 ± 16 years, and 60.7 % of the subjects were < 60-year-old. Most subjects were males (55.3 %), married (73.3 %), village dwellers (58.3 %), and living with their families (93.7 %). The unemployment rate was high (73.3 %) among the subjects, and 59.3 % of them lived with < 2000 New Israeli Shekel (NIS (1 NIS = 0.31 US Dollars)) monthly income. Only 4.7 % did not have any formal education.

As for dialysis-related variables, 65.3 % had been on dialysis for less than four years, and 92 % were undergoing three sessions per week. The majority (86.0 %) received sessions that are less than four hours in duration. Only 4.3 % of the subjects had previous kidney transplantation. More than a third (35.7 %) had ≥ three chronic comorbidities, but most subjects (96.7 %) were on ≥ four chronically used medications. The vast majority (93.7 %) reported a known cause for their ESKD. Most of them (82.0 %) took their medication on their own. Finally, most patients reported having mild pain (73.0 %) and low pain interference (80.3 %). Table 1 presents the demographic and clinical characteristics in detail.

**Table 1:** Participants’ demographic and clinical characteristics

Variable	Frequency (%); *N* = 300
**Age category (years)**	
< 60	182 (60.7)
≥ 60	118 (39.3)
**Gender**	
Male	166 (55.3)
Female	134 (44.7)
**BMI category**	
Underweight	12 (4.0)
Normal	96 (32.0)
Overweight	113 (37.7)
Obese	79 (26.3)
**Residency**	
Refugee camp	30 (10.0)
Village	175 (58.3)
City	95 (31.7)
**Educational level**	
No formal education	14 (4.7)
Primary school	62 (20.7)
Secondary school	81 (27.0)
High school	67 (22.3)
University	76 (25.3)
**Living arrangement**	
Alone	19 (6.3)
With family	281 (93.7)
**Social status**	
Single, divorced, or widowed	80 (26.7)
Married	220 (73.3)
**Employment**	
Unemployed	235 (78.3)
Employed	65 (21.7)
**Household income (per month)**	
< 2000 NIS	178 (59.3)
2000–4999 NIS	109 (36 0.3)
≥ 5000 NIS	13 (4.3)
**Dialysis sessions per week**	
≤ 2	21 (7.0)
3	276 (92.0)
≥ 4	3 (1.0)
**Dialysis vintage (years)**	
< 4	196 (65.3)
≥ 4	104 (34.7)
**Dialysis sessions duration (hours)**	
< 4	258 (86.0)
≥ 4	42 (14.0)
**Previous kidney transplantation**	
Yes	13 (4.3)
No	287 (95.7)
**Known cause for ESKD**	
Yes	281 (93.7)
No	19 (6.3)
**Number of comorbidities**	
None	30 (10.0)
1	77 (25.7)
2	86 ( 28.7)
≥ 3	107 (35.7)
**Number of chronically used medications**	
< 4	10 (3.3)
≥ 4	290 (96.7)
**Pain Severity**	
Mild	219 (73.0)
Moderate	38 (12.7)
Severe	34 (14.3)
**Pain interference**	
Low	241 (80.3)
High	59 (19.7)

Abbreviations: *BMI* body mass index; *ESKD* end-stage kidney disease; *NIS* New Israeli shekel (1 NIS = 0.31 US Dollars)

### Presence, site, and causes of pain

Almost two-thirds (66.3 %) of the subjects reported having chronic pain. The full results on the sites and causes of pain are listed in Table 2. The most commonly reported pain site was the upper and lower limbs (41.2 %). In contrast, the underlying cause for the pain cited by the highest percentage of subjects was the dialysis procedure itself (21.6 %). Causes which were attributed to pain may be due to cannulation, steal syndrome, or cramping [28].

**Table 2:** Presence, site, and perceived causes of pain

Item	Frequency (%); *N* = 300
**Presence of pain**	
Yes	199 (66.3)
No	101 (33.7)
**Pain site**^a^	
Upper and lower limbs	82 (41.2)
Knees	57 (28.6)
Back	53 (26.6)
Shoulders	36 (18.1)
Headache	33 (16.6)
Feet	33 (16.6)
Thighs	21 (10.6)
Abdomen	14 (7.0)
Neck	12 (6.0)
Flanks	4 (2.0)
Chest	2 (1.0)
**Perceived cause of pain**	
Dialysis session	43 (21.6)
Diabetic neuropathy	23 (11.6)
Osteoarthritis	20 (10.1)
Exhaustion	18 (9.0)
General fatigue	16 (8.0)
Osteoporosis	16 (8.0)
Discitis	13 (6.5)
Other or unknown	12 (6.0)
Poor sleep	7 (3.5)
Systemic lupus	7 (3.5)
Constipation	6 (3.0)
Walking	6 (3.0)
Heart failure	5 (2.5)
Peptic ulcer	4 (2.0)
Gout	3 (1.5)

^a^The sum of all percentages is > 100 due to citing multiple pain sites by some subjects

### Management of pain

From 66.3 % of subjects who had pain, 74.0 % tried medications for pain relief. Most subjects (89.4 %) used pharmacotherapeutic measures for pain relief, while the rest tried non-pharmacological measures only, including rest and warm/cold compressors. Some subjects used multiple methods of pain relief at the same time. The degree of relief varied among the subjects, with 26.6 % reported experiencing no relief, and 10.6 % reported experiencing full response and relief. Results on pain relief methods are summarized in Table 3.

**Table 3:** Pharmacotherapys used for pain relief and degree of relief with treatment

Item	Frequency (%); *N* = 300
**Pharmacotherapy**	
Paracetamol	112 (56.3)
Rest	25 (12.6)
Diclofenac sodium	20 (10.1)
Diclofenac potassium	11 (5.5)
Ibuprofen	9 (4.5)
Famotidine, Ranitidine, or Esomeprazole	8 (4.0)
Pregabalin	5 (2.5)
Colchicine	4 (2.0)
Laxatives	4 (2.0)
Coldwater	2 (1.0)
Lornoxicam	2 (1.0)
Chlorzoxazone	2 (1.0)
Warm compressors	1 (0.5)
Scopolamine	1 (0.5)
**Degree of relief**	
0 %	53 (26.6)
10 %	8 (4.0)
20 %	12 (6.0)
30 %	6 (3.0)
40 %	13 (6.5)
50 %	6 (3.0)
60 %	11 (5.5)
70 %	17 (8.5)
80 %	31 (15.6)
90 %	21 (10.6)
100 %	21 (10.6)

### Brief pain invitatory

The mean pain severity score among our subjects was 10.55 ± 10.62, whereas their mean pain interference score was 19.41 ± 18.51. Their medians for the same scores were 8 [0.00-17.75] and 16.5 [0.00–30], respectively. The internal consistency reliability indices of the two subscales through which these two scores were calculated, as calculated by Cronbach’s alpha test, were 0.947 and 0.963, respectively. Additionally, we found a significant positive association between pain interference and severity scores (r was 0.861 with a *p*-value of < 0.001).

### Pain severity score

Table 4 shows the results of variables’ associations with pain severity score. Age (*p* = 0.001), gender (*p* = 0.001), BMI (*p* < 0.001), educational level (*p* = 0.003), employment (*p* = 0.004), income (*p* = 0.009), and number of comorbidities (*p* < 0.001) showed statistically significant association with pain severity score. The rest of the variables that we examined were not significantly associated with this score.

**Table 4:** Pain severity score by subgroups based on demographic and clinical characteristics

Variable	Frequency (%); *N* = 300	Pain severity; Median [Q1-Q3]	*P*-value*
**Age category (years)**			
< 60	182 (60.7)	7 [0–16]	**0.001**^**b**^
≥ 60	118 (39.3)	10 [0–24]	
**Gender**			
Male	166 (55.3)	6 [0–16]	**0.001**^**b**^
Female	134 (44.7)	10 [3-21]	
**BMI category**			
Underweight	12 (4.0)	0 [0–16]	**< 0.001**^**a**^
Normal	96 (32.0)	4 [0–12]	
Overweight	113 (37.7)	6 [0–21]	
Obese	79 (26.3)	16 [8-22]	
**Residency**			
Refugee camp	30 (10.0)	13 [0–23]	0.199^a^
Village	175 (58.3)	8 [0–16]	
City	95 (31.7)	8 [0–22]	
**Living arrangement**			
Alone	19 (6.3)	7 [0–12]	0.412^b^
With family	281 (93.7)	9 [0–18]	
**Educational level**			
No formal education	14 (4.7)	16 [10-26]	**0.003**^**a**^
Primary school	62 (20.7)	10 [5-23]	
Secondary school	81 (27.0)	9 [0–20]	
High school	67 (22.3)	5 [0–16]	
University	76 (25.3)	5 [0–15]	
**Social status**			
Single, divorced, or widowed	80 (26.7)	10 [0–26]	0.092^b^
Married	220 (73.3)	8 [0–16]	
**Employment**			
Unemployed	235 (78.3)	9 [0–16]	**0.004**^**b**^
Employed	65 (21.7)	0 [0–16]	
**Household income (month)**			
< 2000 NIS	178 (59.3)	10 [0–21]	**0.009**^**a**^
2000–4999 NIS	109 (36 0.3)	7 [0–16]	
≥ 5000 NIS	13 (4.3)	0 [0–7]	
**Dialysis vintage (years)**			
< 4	196 (65.3)	8 [0–17]	0.111^b^
≥ 4	104 (34.7)	10 [0–20]	
**Dialysis sessions per week**			
≤ 2	21 (7.0)	0 [0–11]	0.136^b^
3	276 (92.0)	8 [0–18]	
≥ 4	3 (1.0)	15 [12-NA]	
**Dialysis sessions duration (hours)**			
< 4	258 (86.0)	9 [0–18]	0.144^b^
≥ 4	42 (14.0)	7 [0–11]	
**Previous kidney transplantation**			
Yes	13 (4.3)	0 [0–17]	0.327^b^
No	287 (95.7)	8 [0–18]	
**Known cause for ESKD**			
Yes	281 (93.7)	8 [0–18]	0.903^b^
No	19 (6.3)	10 [1-18]	
**Number of comorbidities**			
None	30 (10.0)	1.5 [0–9]	**< 0.001**^**a**^
1	77 (25.7)	5 [0–13]	
2	86 ( 28.7)	9 [0–16]	
≥ 3	107 (35.7)	16 [3-25]	
**Number of chronically used medications**			
< 4	10 (3.3)	6 [0–12]	0.535^b^
≥ 4	290 (96.7)	8 [0–18]	

Abbreviations: *BMI* body mass index; *NA* not available; *ESKD* end-stage renal disease; *NIS* New Israeli shekel (1 NIS = 0.31 US Dollars)

* Bold values denote statistical significance

^a^ Kruskal-Wallis test

^b^ Mann-Whitney U test

### Pain interference score

Table 5 shows in full the results on variables’ associations with pain interference score. Age (*p* < 0.001), gender (*p* = 0.001), BMI (*p* < 0.001), residency (*p* = 0.027), level of education (*p* < 0.001), employment (*p* = 0.001), household income (*p* = 0.005), dialysis vintage (*p* = 0.012), and number of co-morbidities (*p* < 0.001) showed statistically significant association with pain interference score. The rest of the variables that we examined were not significantly associated with this score.

**Table 5:** Pain interference score by subgroups based on demographic and clinical characteristics

Variable	Frequency (%); *N* = 300	Pain interference; Median [Q1- Q3]	*P*-value*
**Age category(years)**			
< 60	182 (60.7)	12 [0–26]	**< 0.001**^**b**^
≥ 60	118 (39.3)	23.5 [9-42.5]	
**Gender**			
Male	166 (55.3)	12 [0–28]	**0.001**^**b**^
Female	134 (44.7)	22 [6-32]	
**BMI category**			
Underweight	12 (4.0)	0 [0-27.5]	**< 0.001**^**a**^
Normal	96 (32.0)	10.5 [0–25]	
Overweight	113 (37.7)	16 [1.5–30.5]	
Obese	79 (26.3)	24 [14-37]	
**Residency**			
Refugee camp	30 (10.0)	29.5 [2-40]	**0.027**^**a**^
Village	175 (58.3)	16 [0–26]	
City	95 (31.7)	14 [2-41]	
**Living arrangement**			
Alone	19 (6.3)	18 [0–22]	0.364^b^
With family	281 (93.7)	16 [0–31]	
**Educational level**			
No formal education	14 (4.7)	27 [18–45]	**< 0.001**^**a**^
Primary school	62 (20.7)	25 [14-37]	
Secondary school	81 (27.0)	17 [0–31]	
High school	67 (22.3)	10 [0–21]	
University	76 (25.3)	8 [0–23]	
**Social status**			
Single, divorced, or widowed	80 (26.7)	18 [2–45]	0.063^b^
Married	220 (73.3)	15.5[0–28]	
**Employment**			
Unemployed	235 (78.3)	18 [2-31]	**0.001**^**b**^
Employed	65 (21.7)	5 [0-19.5]	
**Household income (month)**			
< 2000 NIS	178 (59.3)	18.5 [6 − 3]	**0.005**^**a**^
2000–4999 NIS	109 (36 0.3)	12 [0-28.5]	
≥ 5000 NIS	13 (4.3)	7 [2.5–14]	
**Dialysis vintage (years)**			
< 4	196 (65.3)	13 [0–28]	**0.012**^**b**^
≥ 4	104 (34.7)	21 [5-37]	
**Dialysis sessions per week**			
≤ 2	21 (7.0)	0 [0–11]	0.053^a^
3	276 (92.0)	8 [0–18]	
≥ 4	3 (1.0)	15 [12-NA]	
**Dialysis sessions duration (hours)**			
< 4	258 (86.0)	18 [0–31]	0.083^b^
≥ 4	42 (14.0)	13.5 [0-22.5]	
**Previous kidney transplantation**			
Yes	13 (4.3)	0 [0–17]	0.099^b^
No	287 (95.7)	8 [0–18]	
**Known cause for ESKD**			
Yes	281 (93.7)	16 [0-30.5]	0.992^b^
No	19 (6.3)	17 [9-29]	
**Number of comorbidities**			
None	30 (10.0)	2 [0–23]	**< 0.001**^**a**^
1	77 (25.7)	9 [0-18.5]	
2	86 ( 28.7)	17 [1.5–27]	
≥ 3	107 (35.7)	26 [12-40]	
**Number of chronically used medications**			
< 4	10 (3.3)	11 [0-28.5]	0.575^b^
≥ 4	290 (96.7)	17 [0–31]	

Abbreviations: *BMI* body mass index; *NA* not available; *ESKD* end-stage kidney disease; *NIS* New Israeli shekel

* Bold values denote statistical significance

^a^ Kruskal-Wallis test

^b^ Mann-Whitney U test

### Multiple linear regression analysis

We conducted a multiple linear regression analysis stratified by gender, age, BMI, level of education, employment, income, and the number of other comorbidities, with pain severity score as the dependent variable. Regression analysis revealed that subjects with higher BMI (*p* = 0.018), female patients (*p* = 0.023), and those who had a higher number of chronic diseases (*p* < 0.001) were independently associated with a higher pain severity score. Table 6 summarizes the results of the multiple linear regression model for correlation with the pain severity score.

**Table 6:** Multiple linear regression analysis of associations between variables and pain severity score

Model		Unstandardized Coefficients		Standardized Coefficients	t	*P*-value*	95.0 % Confidence Interval for B		Collinearity Statistics
		**B**	**Std. Error**	**Beta**			**Lower Bound**	**Upper Bound**	**VIF**
1	(Constant)	-0.9	4.47		-0.2	0.842	-9.7	7.91	
	Age	1.5	1.25	0.07	1.2	0.231	-0.96	3.96	1.211
	Gender	2.82	1.24	0.13	2.28	**0.023**	0.39	5.26	1.232
	BMI category	1.66	0.7	0.13	2.37	**0.018**	0.28	3.05	1.16
	Educational level	-0.9	0.52	-0.1	-1.73	0.084	-1.93	0.12	1.279
	Employment	0.81	1.59	0.03	0.51	0.613	-2.33	3.94	1.399
	Income	-1.88	1.07	-0.1	-1.76	0.080	-3.97	0.22	1.236
	Number of co-morbidities	2.81	0.62	0.27	4.55	**< 0.001**	1.6	4.03	1.248

* Bold values denote statistical significance

Findings of the linear regression model, using pain interference score as the dependent variable and the covariates of gender, age, BMI, level of education, employment, income, number of other comorbidities, dialysis vintage, and residency as independent variables indicated that older subjects (*p* = 0.009), subjects with longer dialysis vintage (*p* = 0.008), those with less education (*p* = 0.010), and those who had a higher number of comorbidities (*p* < 0.001) were independently associated with higher pain interference score. Table 7 summarizes the findings of the multiple linear regression model for correlation with the pain interference score.

**Table 7:** Multiple linear regression analysis of associations between variables and pain interference score

Model		Unstandardized Coefficients		Standardized Coefficients	t	*P*-value*	95.0 % Confidence Interval for B		Collinearity Statistics
		**B**	**Std. Error**	**Beta**			**Lower Bound**	**Upper Bound**	**VIF**
1	(Constant)	-11.13	9.09		-1.22	0.222	-29.02	6.76	
	Age	5.58	2.11	0.15	2.64	**0.009**	1.42	9.73	1.22
	Gender	4.06	2.09	0.11	1.94	0.053	-0.06	8.18	1.24
	BMI category	2.32	1.19	0.11	1.95	0.052	-0.02	4.65	1.17
	Educational level	-2.29	0.88	-0.15	-2.59	**0.010**	-4.02	-0.55	1.29
	Employment	1.75	2.71	0.04	0.64	0.520	-3.59	7.09	1.43
	Income	-3.3	1.8	-0.1	-1.84	0.067	-6.85	0.24	1.24
	Number of co-morbidities	4.91	1.05	0.27	4.7	**< 0.001**	2.85	6.97	1.25
	Residency	1.93	1.56	0.06	1.24	0.216	-1.13	5	1.03
	Dialysis vintage	5.34	1.99	0.14	2.68	**0.008**	1.41	9.26	1.03

* Bold values denote statistical significance

## Discussion

In the current study, we examined pain symptoms in patients undergoing HD in Palestine from different aspects using the Brief Pain Inventory assessment tool. This complication is particularly important for ESKD patients since it can severely affect their QOL [17].

Our results showed high pain prevalence among patients with HD, wherein 66.3 % of the subjects in this study reported suffering from pain symptoms. The prevalence of this problem in our study is similar to that reported in other countries such as Egypt (52 %) [17]. Additionally, we found that the average pain severity score was 10.55 ± 10.62, and the average pain interference score was 19.41 ± 18.51. These findings are less pronounced than those of a similar Spanish study where the mean for these scores were 11.39 ± 11.21 and 24.48 ± 23.11, respectively [29].

Concerning socio-demographic characteristics, we found a significant association between age and the two pain scores (i.e., severity and interference), which is consistent with the findings from the previous study [30]. This result could be due to certain barriers that limit the treatment of pain in the elderly, such as the presence of comorbid diseases affecting their mobility or exasperating pain, inability to seek healthcare, and medication costs [30].

In this study, social supports (such as living arrangements, social status, and employment) were not significantly associated with pain. However, spiritual support and family relationships have the potential to improve clinical outcomes and quality of life through decreasing depression levels, increasing access to health care facilities, and improving patients’ compliance with prescribed therapies and nutrition [31].

We also found that females suffer from higher severity and interference of pain. Some studies suggested that the higher the pain sensitivity, the lower pain threshold, higher nerve density, and different psychological experiences of pain and different responses to medications [32].

BMI was another variable that was significantly associated with both pain scores in our study. Several previous studies have concluded that obesity may exacerbate chronic pain by reducing the pain threshold and increasing its sensitivity [33, 34]. Additionally, various endocrine changes that complication obesity may alter the modulation of pain [35].

Lower levels of education were also found to be significantly associated with more pain. The reason behind this finding might be that low education might lead to holding false beliefs about pain and performing maladaptive coping practices. It might also be associated with a lack of essential knowledge about the disease and proper access to pain relief options [36, 37]. Additionally, unemployment and low income, which were also associated with more pain, can put an additional financial strain, limit the opportunities for proper pain management, and decrease social support [38], which might explain our findings that these two variables were significantly associated with worse pain symptoms. Low income can also lead to not being able to afford expensive pain relief methods and decrease patients’ accessibility to healthcare services [39].

The finding that longer dialysis vintage and having multiple comorbidities were significantly associated with higher pain interference might be due to the accompanying lifestyle changes [16, 40, 41].

### Strengths and limitations

This research had several strengths, including its multicenter environment as well as its relatively large sample size. It is also the first study in West Bank, Palestine, to discuss chronic pain and pharmacotherapy used by ESKD patients undergoing HD. Additionally, data was collected through face-to-face interviews, which may have improved the reliability of data. However, the current study faced some limitations. For example, adopting the cross-sectional design in this study precluded making any statements on the causality between variables. Moreover, by adopting the convenience sampling technique, the generalizability of our findings to all HD patients in Palestine might have been somewhat jeopardized. Lastly, the absence of comparison between preexisting conditions (e.g., arthritis, gout, etc.) versus dialysis procedure-related pain limits the interpretation of the burden of disease and therapy options.

## Conclusions

Pain is a prevalent symptom among ESKD patients who are treated with HD in Palestine. Subpopulations that had higher pain severity include the elderly, females, patients with higher BMI, those who have less education, the unemployed, patients living in households with lower incomes, and people living with multiple comorbidities. Our findings provide educational institutions and health care providers who work with ESKD patients with crucial data on pain symptoms among HD patients that can help them achieve their goals of performing better pain documentation and management, achieving higher levels of pain relief, and establishing effective prevention programs. Health officials need to pay more attention to chronic pain in HD patients, develop a clinical and practical plan for managing chronic pain properly, and raise public awareness of ESKD causes and how to halt its progression to decrease healthcare costs and improve patients’ outcomes as much as possible.

## Data Availability

The data that was collected and analyzed for this study are available from the corresponding author on reasonable request.

## Abbreviations

BMI: Body mass index
BPI: Brief pain inventory
CKD: Chronic kidney disease
ESKD: End-stage kidney disease
HD: Hemodialysis
IRB: Institutional review board
NIS: New Israeli shekel
QOL: Quality of life
RRT: Renal replacement therapy
